# Neck muscle function improves after neck exercises in individuals with whiplash-associated disorders: a case–control ultrasound study with speckle-tracking analyses

**DOI:** 10.1038/s41598-024-69136-6

**Published:** 2024-08-13

**Authors:** Gunnel Peterson, David Nilsson, Margaretha Jönsson, Kate Bennett, Anneli Peolsson

**Affiliations:** 1 Centre for Clinical Research Sörmland, Uppsala University, Eskilstuna, Sweden; 2 Unit of Physiotherapy, Department of Health Medicine and Caring Sciences, Linköping University, Campus US, Building 511, 15th Floor, 581 83 Linköping, Sweden; 3 Computational Analytics Support Platform (CASP), Department of Chemistry, Umeå University, Umeå, Sweden; 4 Unit of Clinical Medicine, Department of Health Medicine and Caring Sciences, Occupational and Environmental Medicine Centre, Linköping University, Linköping, Sweden

**Keywords:** Whiplash injury, Ultrasonography, Neck muscles, Spine, Rehabilitation, Physical therapy, Disability, Pain, Musculoskeletal system, Diagnosis, Medical imaging, Outcomes research

## Abstract

A whiplash injury can alter neck muscle function, which remains years after the injury and may explain why symptoms such as persistent pain and disability occur. There is currently limited knowledge about dynamic neck muscle function in chronic whiplash-associated disorders (WAD), and about the extent to which altered muscle function can improve after rehabilitation. Ultrasound can detect mechanical neck muscle function by measuring real-time deformation and deformation rate in the muscles. This method was used for five dorsal neck muscles in participants with chronic WAD versus matched controls in resistant neck rotation. We obtained real-time, non-invasive ultrasound measurements using speckle tracking, multivariate analyses, and mixed-design ANOVA analyses. The results showed altered deformation in the three deepest neck muscle layers, with less deformation area in the WAD group compared to controls in rotation to the most painful side at baseline. Participants in the WAD group performed three months of neck-specific exercises, resulting in improved deformation in the deep neck muscles in WAD and with a similar deformation pattern to controls, and the significant group differences ceased. We reveal new and important insights into the capability of ultrasound to diagnose altered neck muscle function and evaluate an exercise intervention.

## Introduction

Chronic whiplash-associated disorders (WAD) remain challenging to treat, mainly due to the lack of diagnostic tools and insufficient knowledge of the pathophysiology behind persistent neck pain and disability. The neck muscles’ complex motor system^1^ maintains postural control, and interacts with the vestibular, eye, and arm movement control systems^1–3^. Inadequate muscle response after whiplash injury may explain why symptoms such as dizziness, alterations in balance, and difficulties controlling arm moments can occur^4,5^. Muscle control patterns change with pain^6,7^, and can cause reorganization of muscle activation^8,9^. The deep neck muscles are especially important for cervical stability and control of intersegmental motion^10^, but these muscles are difficult to investigate. Methods to improve neck muscle function diagnostics in WAD are therefore a high priority.

Diagnostic ultrasound produces sound waves, and the returning echoes indicate reflection of sound waves at tissue boundaries and from small irregularities in the tissues^11^. The small irregularities in the muscles serve as acoustic markers because they form a unique speckle pattern. Ultrasound real-time video sequences can provide an insight into mechanical neck muscle function^12,13^ through quantitative measurements of muscle deformation (elongation or shortening) and deformation rate (the rate at which the deformation occurs). This method has shown altered mechanical neck muscle function and impaired interaction between muscle layers during repetitive arm movements in individuals with WAD compared to neck-healthy controls^14–16^. The studies^14–16^ investigated isometric neck muscles’ function, i.e. postural control, of the cervical spine. To our knowledge, no study has been conducted to investigate dorsal neck muscle function during dynamic neck rotation in WAD using speckle-tracking analyses. Only a small ultrasound pilot study, including nine patients with WAD and nine controls, has investigated dorsal muscle function during cervical resisted extension^17^. However, only women were included, and the effect of rehabilitation was not investigated.

Conservative treatment, including exercises and advice, is recommended in chronic WAD and should be selected based on the assessed impairments^19^. Fat infiltration in the neck muscles, only seen after whiplash trauma^20^, was modified and neck disability decreased after ten weeks of exercises^21^, but the study only included five individuals with WAD and no control group. Isometric ventral neck muscle function, evaluated with ultrasound speckle-tracking analyses, was improved in chronic WAD after 12 weeks of neck-specific exercises (NSE)^22^. NSE also improved neck disability and neck pain compared to general exercises^23^ in chronic WAD. Moreover, NSE with internet support and four visits to a physiotherapist (NSEIT) was non-inferior to NSE^24^, but exercise interventions for WAD are still being debated due to conflicting results^18,25, 26^. Rotation of the head is painful and limited in WAD^27^, and we need to gain knowledge about how neck muscle layers are activated and whether NSE/NSEIT can improve muscle function during neck rotation.

The aims of the present study were: (a) to compare deformation and deformation rate in five dorsal neck muscles (trapezius, splenius, semispinalis capitis, semispinalis cervicis, and multifidus in chronic WAD versus age- and sex-matched healthy controls during dynamic resistant neck rotation; and (b) to evaluate deformation and deformation rate in the five dorsal neck muscles in WAD after three months of neck-specific exercises. We hypothesized an altered mechanical neck muscle function, defined as altered deformation and deformation rate, in WAD compared to healthy controls and improvement in mechanical neck muscle function after three months of exercise.

## Results

**Figure 1:**
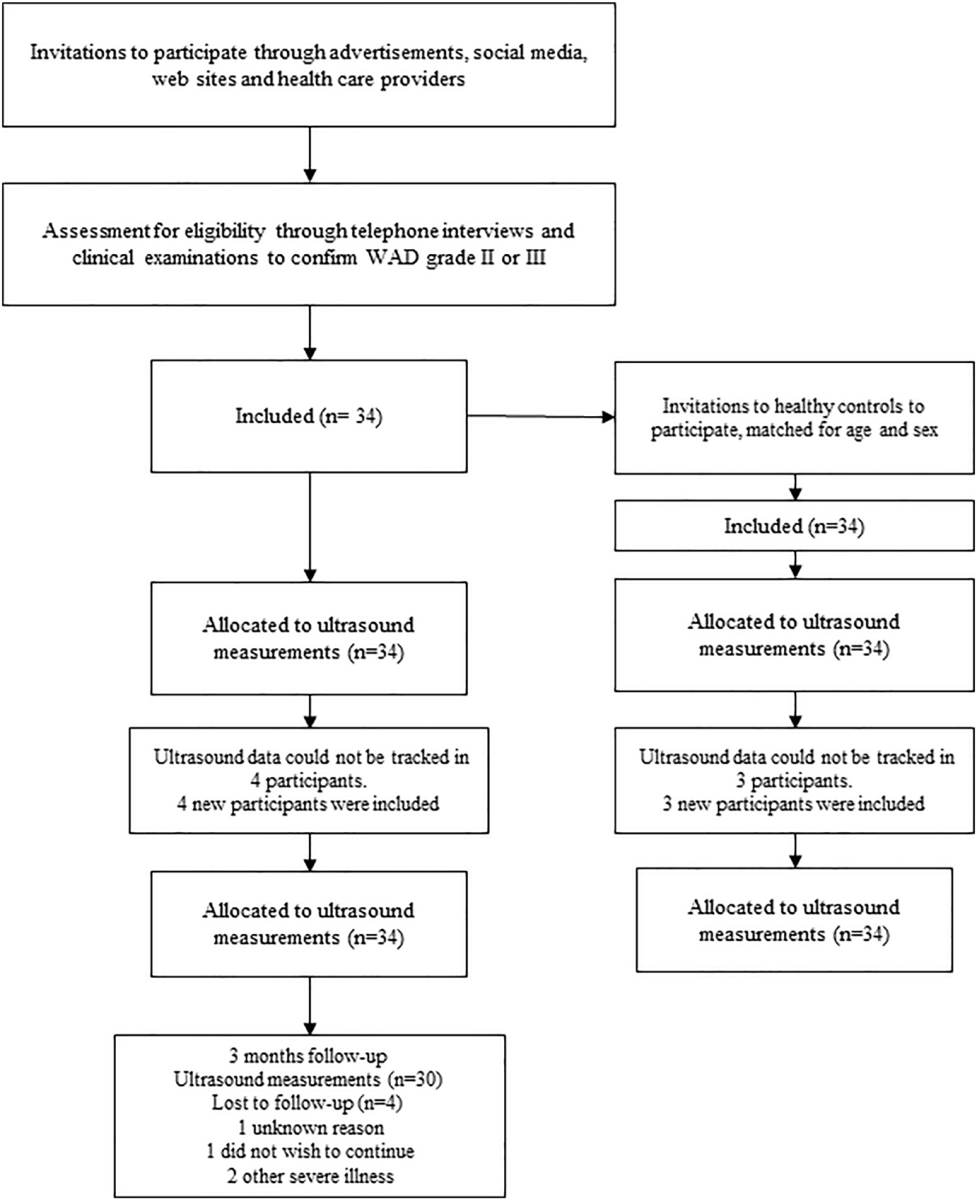
Flow chart.

Thirty four individuals—26 women and eight men (mean age 41.3 years; SD 10.6)—with persistent WAD (mean time since whiplash injury 26.4 months; SD 13.4), average pain intensity during the last week of 46.0 mm (SD 20.1) on the visual analogue scale (VAS; 0 = no pain, 100 = worst imaginable pain), and neck disability rated as 39% (SD 13.7) on the Neck Disability Index (NDI; 0% = no disability, 100% = highest score for disability) were recruited consecutively for ultrasound investigation (Table 1). Thirty individuals from the WAD group received three months of neck-specific exercises (Fig. 1).

**Table 1:** Baseline characteristics of participants with whiplash-associated disorders (WAD) and healthy controls.

	WAD (n = 34)	Control (n = 34)	*P*-value
Age, mean ± SD	41.3 ± 10.6	41.7 ± 10.7	0.87
Sex, female, n (%)	26 (76%)	26 (76%)	1.0
BMI, mean ± SD	24.1 ± 3.6	24.8 ± 3.4	0.32
Months since injury, mean ± SD	26.4 ± 13.4	NA	
WAD grade, grade III, n (%)	17 (50%)	NA	
Physical activity level, median (IQR)	3.0 (2.0 to 3.25)	4.0 (3.0 to 4.0)	< 0.01
Neck pain average last week (VAS 0–100), mean ± SD	46.0 ± 20.1	1.6 ± 2.8	< 0.001
Neck pain, current (VAS 0–100), mean ± SD	42.0 ± 21.3	0.03 ± 0.17	< 0.001
Neck muscle fatigue (Borg CR 0–10), median (IQR)	4.0 (3.0 to 6.2)	0.0 (0.0 to 0.1)	< 0.001

*WAD* whiplash-associated disorders; *SD* standard deviation; *BMI* body mass index; physical activity level during the previous 12 months (1 = inactivity, 2 = low activity, 3 = moderate activity, 4 = high activity); *IQR* inter-quartile range; *VAS* visual analogue scale; Borg CR 0–10: neck muscle fatigue (0 = no fatigue, 10 = extremely strong fatigue).

The WAD group was compared to 34 healthy controls, matched for age (mean age 41.7 years; SD 10.6) and sex, with a pain intensity of 1.6 mm (SD 2.8) and NDI 1.1% (SD 1.6) (Table 1).

There were no significant differences between the groups in test time for the right (WAD; mean 3.94 s [SD 0.75], controls; 4.21 s [0.27], *p* = 0.07) or left neck rotation (WAD; mean 4.09 s [SD 0.39], controls; 4.20 s [0.17], *p* = 0.14). Mean and standard deviation in muscle deformation and deformation rate in the five neck muscles, NDI, neck pain, and neck muscle fatigue in the WAD and control groups can be seen in Supplementary File 1, Tables SI and S2.

### Comparisons of deformation area in the WAD and control groups, rotation to the right

**Figure 2:**
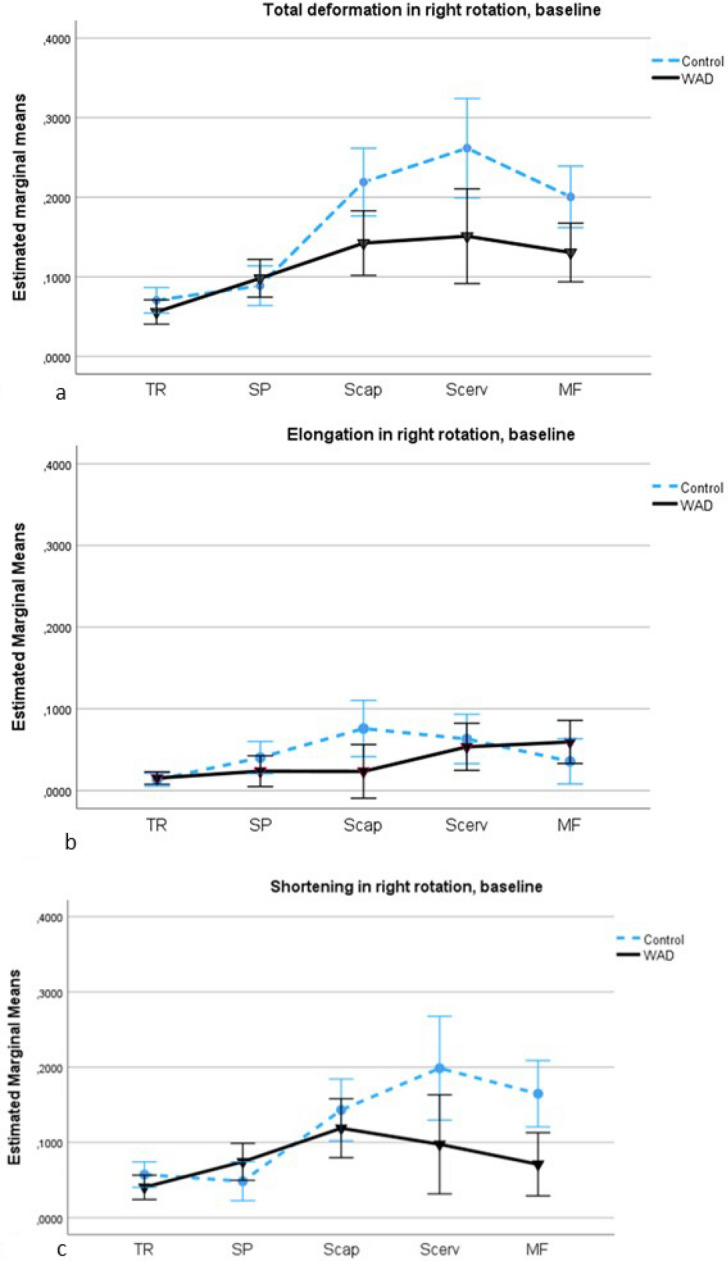
**(a–c)** Deformation area in right rotation in the WAD and control groups at baseline. (**a**) Total deformation area represents the sum of elongations and shortenings of the muscles; (**b**) Deformation area elongation; (**c**) Deformation area shortening. The estimated marginal mean values (deformation area) with a 95% confidence interval (CI) in the five dorsal neck muscles (TR: trapezius; SP: splenius; Scap: semispinalis capitis; Scerv: semispinalis cervicis; MF: multifidus) in the WAD (black line, mean value ) and control groups (blue line, mean value blue dot).

There was a significant main effect of deformation between the groups (all five muscles [F(1,60) = 12.4, *p* < 0.001, ηp^2^ = 0.17]), with higher deformation in the control group compared to the WAD group (mean: 0.053; 95% confidence interval [CI] 0.023–0.0820). There was a significant interaction effect of muscles by group in total deformation (F[2.7,161] = 3.47, *p* < 0.009, ηp^2^ = 0.05). The post-hoc pairwise contrasts showed significant differences in total deformation between the superficial muscles (trapezius, splenius) and the middle muscle semispinalis capitis (F[1,60] = 6.8, *p* = 0.012, ηp^2^ = 0.10), and between the superficial muscles and the deepest muscles (semispinalis cervicis, multifidus) (F[1,60] = 9.6, *p* = 0.003, ηp^2^ = 0.14) (Fig. 2a). Compared to the control group, the WAD group had significantly less elongation in the middle muscle (F[1,60] = 5.8, *p* = 0.019, ηp2 = 0.09), and less shortening was seen in the deepest muscles (F[1,60] = 10.5, *p* = 0.002, ηp2 = 0.15) (Fig. 2b,c).

### Comparisons of deformation area in the WAD and control groups, rotation to the left

**Figure 3:**
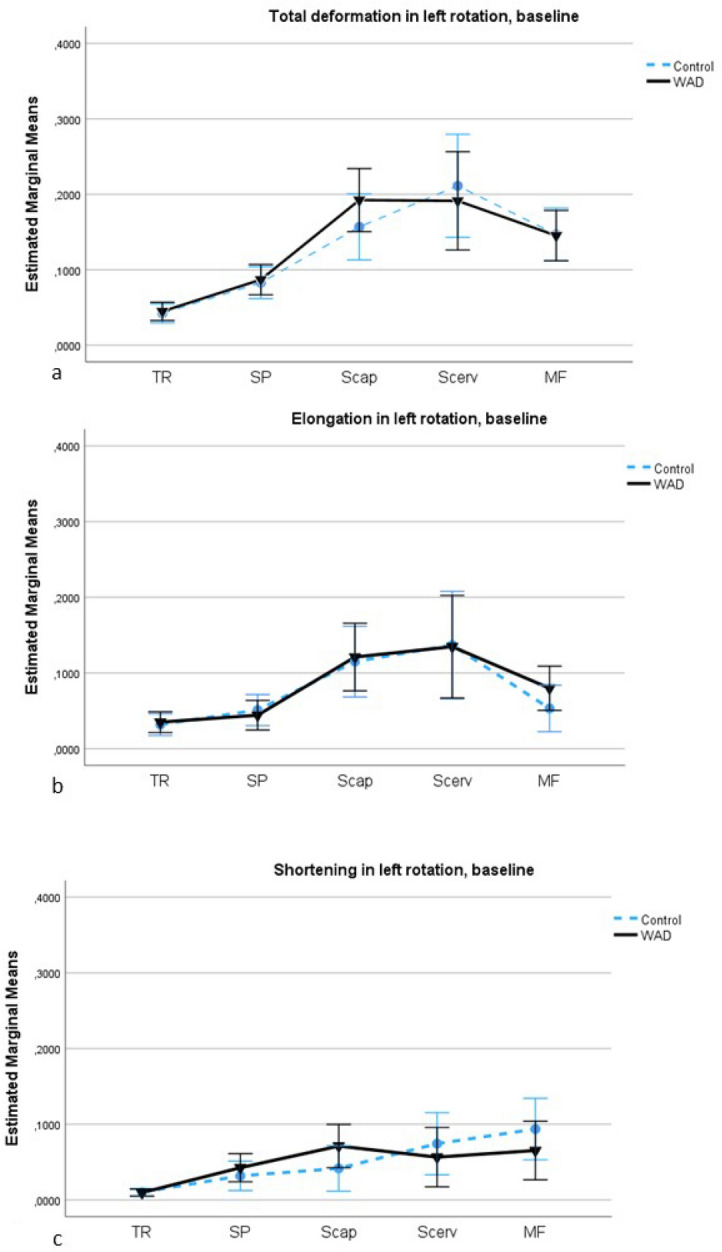
**(a–c)** Deformation area in left rotation in the WAD and control groups at baseline. **(a**) Total deformation area represents the sum of elongations and shortenings of the muscles; (**b**) Deformation area elongation; (**c**) Deformation area shortening. The estimated marginal mean values (deformation area) with a 95% confidence interval (CI) in the five dorsal neck muscles (TR: trapezius; SP: splenius; Scap: semispinalis capitis; Scerv: semispinalis cervicis; MF: multifidus) in the WAD (black line, mean value ) and control groups (blue line, mean value blue dot).

There was no significant main effect of deformation between the groups (all five muscles [F(1,60) = 0.1, *p* = 0.79, ηp^2^ = 0.001]) or interaction effect of muscles by group (F[2.2,132.8] = 0.6, *p* = 0.58, ηp^2^ = 0.009) in left neck rotation. The pairwise contrasts showed no significant interaction effect of muscle by group (Fig. 3a–c; *p* > 0.23). Supplementary File 2 (Figure SI) shows examples of the whole deformation curve during right and left rotation in the WAD and the control groups.

### Comparisons of deformation rate in the WAD and control groups

There was no significant main effect of deformation rate between the groups in right (all five muscles, F[1,60] = 0.6, *p* = 0.43, ηp^2^ = 0.01) or left rotation (F[1,60] = 0.4, *p* = 0.53, ηp^2^ = 0.01) at baseline. The pairwise contrasts showed no significant interaction effects of muscle rate by group in right (F[3.2,192] = 2.5, *p* = 0.055, ηp^2^ = 0.04) or left rotation (F[2.6,159] = 1.2, *p* = 0.30, ηp^2^ = 0.02).

### Comparisons of deformation area in the WAD group after three months of neck-specific exercise versus baseline data in the control group, rotation to the right

**Figure 4:**
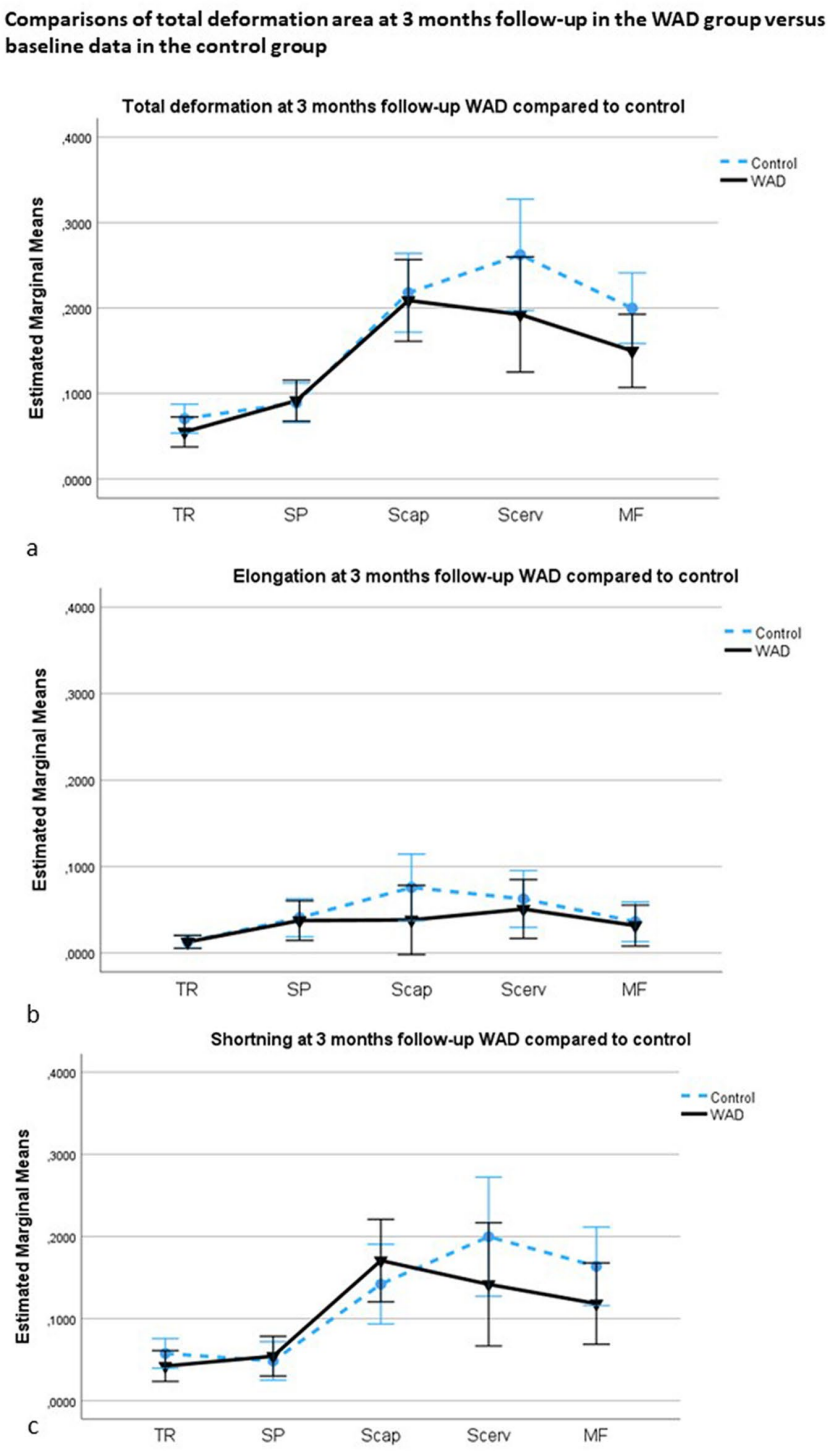
**(a–c)** Comparisons of deformation area in the WAD group after three months of neck-specific exercise versus baseline data in the control group, rotation to the right. (**a**) Total deformation area represents the sum of elongations and shortenings of the muscles; (**b**) Deformation area elongation; (**c**) Deformation area shortening. The mean values with a 95% confidence interval (CI) in the five dorsal neck muscles (TR: trapezius; SP: splenius; Scap: semispinalis capitis; Scerv: semispinalis cervicis; MF: multifidus) in the WAD (black line, mean value ) and control groups (blue line, mean value blue dot).

There was no significant main effect of deformation between the groups after three months of neck-specific exercises in WAD (all five muscles, F[1,55] = 2.7, *p* = 0.10, ηp^2^ = 0.05) or interaction effects of muscle deformation by group (F[2.6,148] = 1.2, *p* = 0.32, ηp^2^ = 0.02). The pairwise contrasts showed no significant interaction effect of muscle by group (WAD and control) between the superficial muscles (trapezius, splenius) and semispinalis capitis (F[1,55] = 1.6, *p* = 0.21, ηp^2^ = 0.02), or between trapezius/splenius, and the deepest muscles (semispinalis cervicis, multifidus) (F[1,55] = 0.1, *p* = 0.70, ηp^2^ = 0.003) (Fig. 4a–c).

### Within-subject effects in the WAD group from baseline to three-month follow-up

There were significant within-subject effects in the WAD group between baseline and three months follow-up (F[1,69] = 5.1, *p* = 0.03, ηp^2^ = 0.16) in deformation in the neck muscles (mean: 0.025; 95% confidence interval [CI]: 0.002–0.049). The middle muscle semispinalis capitis showed significantly higher deformation area at three-month follow-up compared to baseline (*p* = 0.013).

### Multivariate analyses

The initial multivariate analyses confirmed the results from the mixed design ANOVA analyses at baseline and three-month follow-up. Deformation was significantly altered in the three deepest neck muscle layers, with less deformation in the WAD group compared to the control group (semispinalis capitis [*p* = 0.011], semispinalis cervicis [*p* = 0.013], and multifidus [*p* = 0.011]) during right neck rotation, and no significant differences were seen at three-month follow-up (semispinalis capitis [*p* = 0.787], semispinalis cervicis [*p* = 0.142], and multifidus [*p* = 0.099]).

During left neck rotation, the multivariate analysis showed no difference in deformation between the WAD group and the control group for all five muscle groups (trapezius [*p* = 0.772], splenius [*p* = 0.785], semispinalis capitis [*p* = 0.246], semispinalis cervicis [*p* = 0.6749], and multifidus [*p* = 0.950]). This was in agreement with the mixed design ANOVA analyses, which revealed no significant differences between the WAD and control groups.

### Neck disability and pain intensity

The WAD group had significant improvements in neck disability (NDI; mean 9.5, SD 8.5, *p* < 0.001), neck pain (VAS; mean 18.9, SD 25.2, *p* < 0.001), and neck muscle fatigue after tests (Borg CR-10; mean 2.0, IQR 0.0 to 3.1, *p* < 0.001) from baseline to three-month follow-up (Supplementary File 1, Table SI).

## Discussion

The results showed significantly altered neck muscle function in resistant cervical rotation to the most painful side in individuals with persistent WAD grades II and III compared to age- and sex-matched healthy controls. After three months of neck-specific exercises, the differences between the WAD and control groups were no longer significant and the WAD group showed significant improvements over time. The results may indicate that altered mechanical neck muscle function during cervical rotation in chronic WAD can be investigated with ultrasound and speckle-tracking analyses, and can be used to evaluate exercise programs.

Our results revealed less total deformation area in the three deepest muscle layers (semispinalis capitis, semispinalis cervicis, and multifidus) in neck rotation to the right in the WAD group compared to controls. Trapezius and splenius showed less total deformation in right (ipsilateral) rotation at baseline in both the WAD and control groups compared to the deeper muscles. The two deepest muscles showed less shortening deformation in right (ipsilateral) rotation in the WAD group. These results are not consistent with earlier studies on specimens or with computer modelling, suggesting contralateral activation in the deepest muscles^28,29^ and in trapezius^28^ related to the muscle’s moment arm, and that splenius^28^ is the greatest contributor to ipsilateral rotation. However, Bexander et al.^30^ investigated EMG activity in multifidus with different eye positions during neck rotation in healthy individuals and found no differences in activation related to degree of rotation or rotation direction. In a later study^31^, performing the same test in WAD individuals, activation in multifidus was altered and lower in WAD individuals^31^ in contrast to the results for healthy individuals^30^. Moreover, an unstable head movement task showed greater EMG activity in multifidus compared to the superficial neck muscles in healthy individuals. When the difficulty of the task increased there were no or small differences in the deep neck muscles’ EMG activation unlike the superficial muscle activity that increased with increasing task difficulty^32^. The EMG results indicate that the deep muscles support the cervical spine to produce a stable base during movement of the head^30,32^, but it is challenging to interpret speckle-tracking ultrasound investigations compared to other methods^28–32^.

A speculative explanation for our results is that ultrasound measurement during a small rotation task to 20° in a standing position with low resistance may detect the control of intersegmental motion to stabilize the cervical spine. The shortening of the deep neck muscles in healthy individuals may show this control motion in right rotation, which is not seen in the WAD group. Trapezius and splenius may contribute more to rotation over 20° or at higher resistance, and could therefore have lower deformation in the present study.

We investigated the right dorsal neck muscles during both right and left rotation, but surprisingly there were no significant differences between the WAD and control groups in left rotation. The different deformation pattern in left rotation compared to right rotation, see Figs. 2 and 3, may explain the results, showing less shortening deformation, especially in the three deepest neck muscles, in left rotation compared to right rotation in the control group. The muscles’ moment arms and force-generating capacity change related to different cervical movements^29,33, 34^. In axial rotation is the moment-generating capacity lower in multifidus with contralateral rotation (related to decreases in moment arm) and greater with ipsilateral rotation (because of increases in both moment arm and force)^34^. Thereby, the increases in moment arm and force in multifidus in right (ipsilateral) rotation may enhance load and strain to the cervical facet joints and capsule and cause more pain and altered muscle function in right rotation in the WAD group when the muscle was shortened. The multifidus muscle attaches directly to the facet capsule in the cervical spine^34^, and the cervical facet joints and capsule have been proposed to be a source of chronic pain following whiplash injury^35^. Shortening deformation in semispinalis capitis and cervicis muscles may also increase load to painful structures in right rotation and affect these muscle’s function. Pain alters muscle function^7,8^, and the differences in neck kinematics and load^29,33, 34^ may explain why the mechanical neck muscles’ function (deformation) was significantly changed between the WAD and control groups in right (ipsilateral) rotation but not in left (contralateral) rotation.

Knowledge of the neck muscle anatomy, the neck muscle architecture, and the biomechanical response of the neck musculoskeletal system is based on cadaver dissection, biomechanical computer modelling^29,33, 34^, and experimental testing using electromyography (EMG) in isometric neck muscle activation^36,37^. During the last decade, EMG studies of healthy controls have improved our knowledge of the complex neck muscle function^7,8, 38^, showing large intra- and inter-subject variations. Fice et al.^38^ compared electric stimulation of neck muscles versus active isometric contraction. The active isometric contraction in semispinalis capitis had only a small component of ipsilateral rotation^37^, and the intrasubject variability was large at low activation intensity in isometric contraction in the splenius and semispinalis capitis muscles^38^. The active isometric contraction was related to the combination of numerous muscles, and were different compared to the function of the muscles when they were electrically stimulated. Other studies^8,9^ reported that semispinalis cervicis assists rotation of the head when acting unilaterally^8^, and there was a subject-specific variation in superficial muscles’ adaptation to pain^7^.

In this study, ultrasound measurement shows the longitudinal mechanical deformation of the neck muscles, and is different from EMG, which measures the neuromuscular activation of muscle fibers. We cannot therefore conclude that shortening of the muscles captured using speckle tracking indicates muscle contraction. However, for more than a decade, longitudinal deformation measurements using speckle-tracking analyses have been extremely useful for accurate detection of alterations in the heart muscle^39,40^. Ultrasound with speckle-tracking analyses showed a moderate to high relationship between deformation and force (maximal voluntary contraction [MVC]) in neck^13^ and arm/shoulder muscles^41^. In future, the non-invasive ultrasound method may also be a useful tool in healthcare for investigating the function of skeletal muscles.

The WAD group improved in neck disability, pain, and neck muscle fatigue after three months of neck-specific exercises. The results in this study strengthen the theory about the connection between pain, disability, and impaired deep neck muscle function. However, a cause-and-effect relationship cannot be established between mechanical muscle function and pain in the present study.

Trial heterogeneity, small sample size, and different methods make it difficult to draw conclusions about neck muscle activation or mechanical function between studies. To our knowledge, only two studies have evaluated dynamic neck movement using speckle-tracking analyses^42,17^. Bjorklund et al. showed the highest deformation in contralateral semispinalis capitis in quadruped standing in individuals without neck pain^42^, but the test position and resistance were different from the tests in the present study. A significantly different pattern of deformation was seen in trapezius and multifidus in nine women with WAD compared with healthy controls in a loaded extension task^17^, but the study did not evaluate any exercise intervention.

### Limitations

The present study has several limitations. Ultrasound measurements of local deformation in muscles have been validated against force measurements^13,41, 43^, but more studies are required to validate the method during dynamic exercises. Moreover, ultrasound with speckle-tracking analyses to detect neck muscle deformation needs further investigation for validity, reliability and normative reference values before it can be used in the clinical setting. The repeatability of the probe placement could also have been limited due to the lack of significant anatomical landmarks, although the C4 spinous process and vertebral column were used as reference points. The ultrasound probe was placed in a longitudinal position, which entailed the neck muscles being scanned at different angles in relation to the muscles’ fiber. This has an impact when comparing the five muscles, as detectible elongation and shortening are related to muscle fiber direction. However, this limitation did not affect the comparison between the WAD and control groups. The physiotherapists performing the ultrasound measurements were not blinded for group allocation, that possibly could have biased the measurement and results. To minimize the risk of biased results the test followed a strict protocol. Moreover, during the test, no information of speckle-tracking results can be seen and nor did they affect the measurements as speckle-tracking analyses were post-processed. The researcher performing the post-process speckle-tracking analyses was blinded to group. We found significant differences in deformation at group level, but the shortening and elongation deformation in the neck muscles showed large inter-subject variability in both groups, as seen in Supplementary File 2. However, in healthy controls, the two deepest muscles were shortened in most of the individuals, and with greater deformation in semispinalis capitis in right rotation compared to the WAD group.

## Conclusions

In conclusion, we have shown that individuals with chronic WAD have significantly less deformation in the three deepest neck muscles in ipsilateral right rotation compared to healthy controls. In addition, after three months of neck-specific exercises, deformation in these muscles was improved and there were no significant differences between the WAD and control groups. Moreover, these promising results in improved neck muscle function, pain, disability, and muscle fatigue included those with longstanding neurological signs after whiplash injury, WAD grade III. However, to confirm the results, larger randomized studies with control groups are required. The non-invasive ultrasound method can be used in the assessment and diagnosis of patients following whiplash trauma, but needs to be evaluated further.

## Methods

### Design and setting

**Figure 5:**
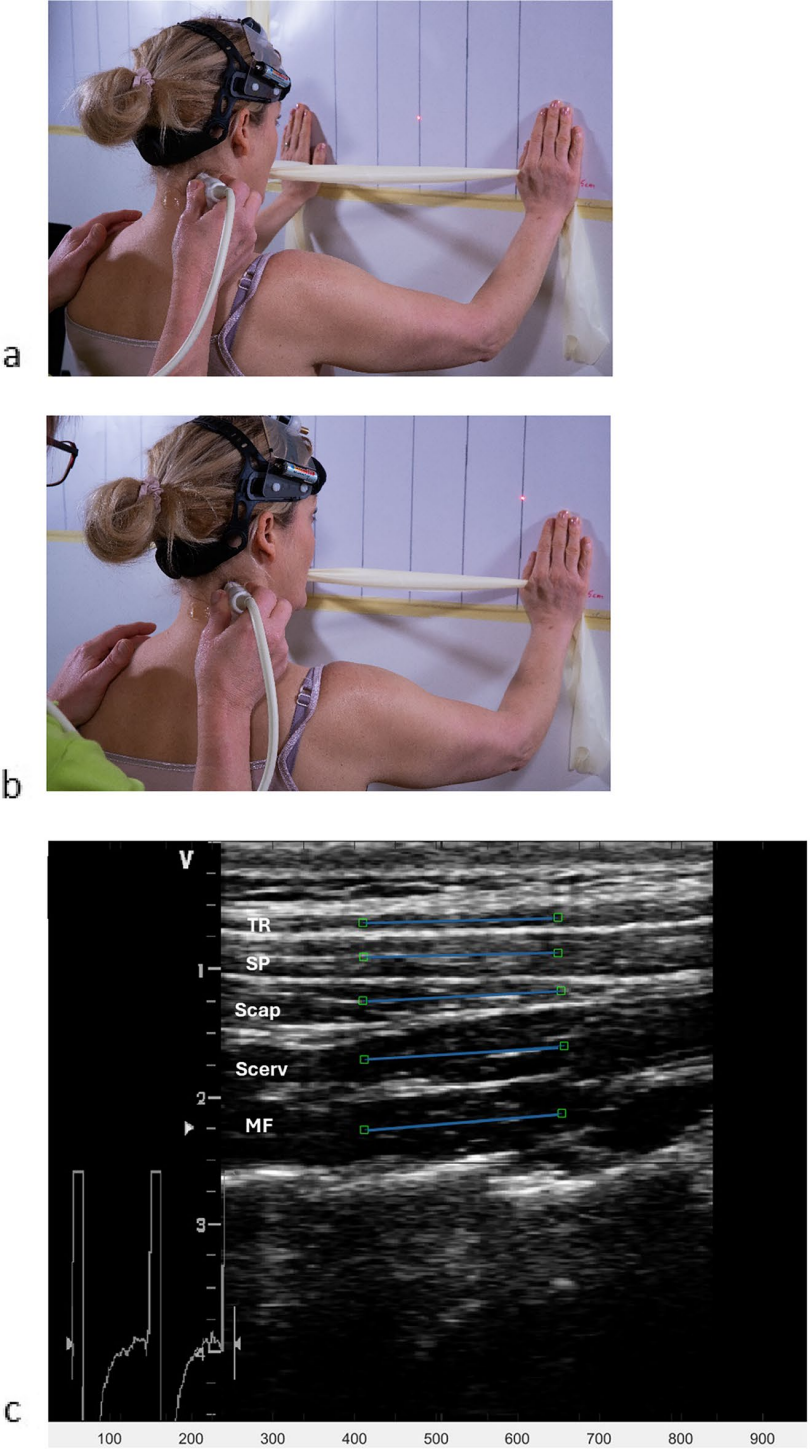
**(a)** Starting position for the rotation test. Five lines were marked on the wall: starting position (midline), two lines to the right (first line 10-degree rotation, second line 20-degree rotation), and two lines to the left (first line 10 degrees, second line 20 degrees). A self-chosen neutral head position was the starting position. The laser cursor at the helmet was adjusted to the starting line (midline marked on the wall). The participant held a rubber band with light resistance (TheraBand Tan) gently between their teeth in line with their mouth. The rationale for using the task in the present study was to investigate if resistance neck rotation could detect altered neck muscle function in chronic WAD and be used to evaluate muscle function after an exercise programme. In our clinical experience, light resistant neck rotation improves neck pain and disability in many chronic WAD patients, indicating that this exercise improves neck muscle function which may be important for good outcomes. (**b)** Twenty-degree rotation to the right. (**c)** The ultrasound image shows the longitudinal projection with the superficial trapezius (TR) at the top, followed by splenius (SP), semispinalis capitis (Scap), semispinalis cervicis (Scerv), and multifidus (MF). A region of interest (ROI) was manually placed in the first frame of the video sequence of the muscle, measuring the deformation (elongation and shortening) and the deformation rate (how fast the shortening and elongation occur) using speckle-tracking analyses. One 15 mm ROI was placed in each muscle (indicated as a blue line with a square at each end).

In this case–control study, we investigated neck muscle function in individuals with chronic WAD compared to pain-free controls, defined as average pain during the last week VAS < 4 mm^44^, with no neck disability. The participants were recruited from nine county council areas in Sweden between October 4, 2018 and December 16, 2021 (Fig. 1). The study was approved by the Regional Ethics Review Board in Linköping, Sweden (ref. 2016/135–31 and 2017/556–32), and was conducted in accordance with the Declaration of Helsinki. Informed written consent to participate and to publish information was obtained from all trial participants. The protocol was registered before data collection started^45^ at Clinicaltrial.gov (Protocol ID: NCT03664934, first posted date 11/09/2018). Informed consent was obtained to publish the images (Fig. 5a,b) in an online open access publication.

### Participants

Study inclusion criteria were persistent symptoms associated with a whiplash injury six months to five years prior to study entry and having exhibited neck symptoms within the first week after the car accident; WAD grade II (neck pain and musculoskeletal signs) or III (neck pain, musculoskeletal signs, plus neurological signs); age 18–63 years; persistent neck pain rated greater than 20 mm on a VAS and neck disability greater than 20% measured with the NDI; neck pain on the right side of the neck, or bilateral neck pain if the right side is the most painful; the individual is right-handed; and daily access to a computer/smartphone/tablet and the internet. Exclusion criteria were signs of traumatic brain injury at the time of whiplash injury; known or suspected serious pathology; previous fracture or luxation in the cervical spine; contraindication to exercise; neuromuscular diseases; rheumatologic disease; previous neck pain causing more than one month of sick leave in the year before the whiplash injury; severe mental illness; and current alcohol or drug abuse.

Inclusion criteria for healthy controls were age and sex matched to the individuals with WAD; right-handedness; no present or past neck pain; no trauma to the neck or head, including whiplash injury; no neck pain defined as average pain during the last week VAS < 4 mm^44^, no low back pain; no rheumatologic or neurologic disease; and no generalized myalgia.

### Recruitment

Information about the study was provided via healthcare providers, reports in newspapers, social media, and the university’s website. Interested participants contacted the research team through the project’s website. They completed a short survey on the website, and a project team member (physiotherapist) conducted a telephone interview to further verify their medical history and eligibility for inclusion in the study. The last step in recruiting participants with WAD was a physical examination conducted by a test leader (physiotherapist). If the criteria for study participation were met, the project team member arranged an appointment for the ultrasound test. Healthy individuals were recruited consecutively among friends, family, and staff, and via social media. A project team member (physiotherapist) conducted a telephone interview to further verify eligibility for inclusion in the study as healthy controls. Consecutive inclusion was used to include 30 participants with WAD who completed three months of follow-up.

### Procedure

#### Ultrasound measurement

All measurements were conducted at Linköping University. The dorsal neck muscles were evaluated with a B-mode, 2-D ultrasound Vivid-I scanner (GE Healthcare, Horten, Norway) connected to a hand-held 12 MHz linear transducer (38 mm) at 50 frames/s. Five dorsal neck muscles (upper trapezius, splenius capitis, semispinalis capitis, semispinalis cervicis, and multifidus/rotatores) were recorded at the 10th repetitive neck rotation, first to the right and then to the left. The neck rotations were standardized in terms of range and speed of movements. Each participant was asked to stand in a comfortable upright position with their feet behind a line marked on the floor (Fig. 5a,b).

A self-chosen neutral head position was established as the starting position, and a laser cursor on a helmet worn by the participant was adjusted to the starting line (midline marked on the wall). The participant held a rubber band with light resistance (TheraBand Tan) gently between their teeth, with their hands on the wall (outside the second line to the right and left) in line with their mouth. The rubber band with light resistance was used to investigate whether deformation and deformation rate are altered in a dynamic exercise that may have potential as a test to measure dynamic neck muscle function in the future in individuals with WAD. The experimental procedure consisted of two parts. First, the neck was rotated from a neutral head position 20 degrees to the right (second line to the right) and back to midline, ten times.

After a short rest (60 s), the procedure was repeated to the left, and the neck was rotated to the left and back to the midline ten times. A metronome was set at 30 beats/minute to maintain a steady pace. Instructions were given to the participants as follows: “Listen to the beat of the metronome. On the first beat, rotate your neck to the right to reach the second line on the second beat, and return to neutral on the third beat. Continue rotation to the right with a steady pace, and reach the line to the right and return to the midline on each beat.” To ensure familiarization with the test, the individual practiced three neck rotations to the right and three neck rotations to the left without the rubber band. Ultrasound video sequences were obtained during the tenth neck rotation to the right and left. All ultrasound measurements were performed with the transducer held in a longitudinal position at the level of the fourth cervical vertebra (C4) on the right side of the neck, which was identified by palpation of the C-4 spinous process. Two experienced physiotherapists carried out the ultrasound test. One performed the ultrasound examination and the other assisted the ultrasound examiner, instructed the participants, and used a custom-made contact switch to synchronize data between neck rotation and ultrasound measurements. The contact signal was recorded using the ultrasound machine in the channel for the ECG system, to provide a cue for synchronizing data between the ultrasonography and the starts (midline), at 20-degree rotations and stops (back to midline) for each neck rotation. The physiotherapists performing the ultrasound measurements were not blinded to group allocation. The researcher performing post-process speckle-tracking analyses was blinded.

### Other measurements

The participants completed a baseline questionnaire prior to ultrasound imaging, asking about age, gender, average pain intensity experienced during the previous week, and current pain (visual analogue scale [VAS], 0 = no pain, 100 = worst imaginable pain)^46^ and neck disability (neck disability index [NDI], 0% = no disability, 100% = highest score for disability)^47^. WAD grade, neck muscle fatigue before and after the test (Borg CR-10 scale: 0 = no fatigue, 10 = extremely strong fatigue)^48^, and activity level (activity index: 1 = inactivity, 2 = low activity, 3 = moderate activity, 4 = high activity)^49^ were also recorded (Table 1).

#### Neck-specific exercise

Initially, patients were randomized to 12 weeks of neck-specific exercise distributed in two different ways: NSEIT with four visits to the physiotherapist (n = 25) or NSE with visits twice a week to the physiotherapist (n = 5). All patients recruited to the study after February 2020 were assigned to the NSEIT group because of the Covid-19 pandemic and limited capacity in the healthcare service. In the present study, the NSE and NSEIT groups was treated as one WAD group, as the main outcome data from a randomized controlled trial reveals the outcome to be equal^24^. Previously, NSE was shown to have a significantly better effect compared to remaining on a waiting list^50^ or general physical activity^23^. All participants in both groups received the same information and the same neck-specific exercise program. The first exercises in the program were targeted at facilitating and activating the deep neck muscle layers, with individual progression of endurance exercises within the patient’s symptom tolerance.

In the NSEIT group, information and the exercise program were delivered via the internet. The participants in the NSE group received their information when visiting the physiotherapist, and received the exercise program in printed form.

The first session for the NSEIT group included a clinical examination and an introduction to the first exercises. The follow-up sessions (weeks 2, 3, and 7) involved new exercises, progression of exercises, and follow-up to ensure correct performance. Patients had access to the internet-based program (produced for research in-house by AP and GP) on a website, with information, photos, and videos of all the exercises, and with clear stepwise progression.

The patients in the NSE group received the same information and exercise program, but delivered by the physiotherapist at the clinic. Initially, the exercises were also performed daily at home.

Patients in both groups were encouraged to continue training on their own two or three times a week after the 12-week intervention, in accordance with the 2017 World Health Organization guidelines, and to include NSEs in their training program. Both interventions have been clearly described previously^24^.

### Speckle tracking

Kanade, Lukas, and Tomasi developed the algorithm for the speckle-tracking method^51,52^, which was further improved by Farron et al.^53^. The speckle-tracking methodology was implemented with an in-house software program written in Matlab (Matlab 2018b, Mathworks, Natick, MA). A region of interest (ROI) was manually placed in the first frame of the video sequence for the muscle, making it possible to track the unique speckle pattern frame by frame through the video sequence. The analyzer was blinded to group affiliation, as all ultrasound video sequences were coded during the post-process analysis. The ROI consisted of a large number of measuring points, and the frame-to-frame displacement could be obtained with a least squares fit assuming a linear strain model. Accordingly, when the speckle pattern changes length with muscle activity, so does the length of the ROI. Elongation or shortening of the muscles, i.e. *muscle deformation*, was calculated as the percentage change from the original length of the ROI compared to rest, and was expressed as % deformation. The *muscle deformation rate* was expressed as the amount of deformation per time unit (% deformation/s). An ROI (15 mm) was positioned longitudinally to the muscle fibers in each muscle. To estimate muscle deformation, the areas on the deformation curves were calculated (Supplementary File 2, Fig. S2). The trapezoidal rule (Eq. 1)—where A is the area, t is the time between samples and y_n_ is the current ROI position at sample point n—was used as the basis for area calculation. The equation was modified to deal with intersections with the 0% line. Linear interpolation was used to estimate additional sample points with adjusted t-values at intersections with the 0% line. The areas below and above the 0% line could thereby be separated.

1 $$A = \frac{t}{2}{\text{(y1 + 2y2 + 2y3 + }} \cdots {\text{ + 2yn - 2 + 2yn - 1 + yn)}}$$

To provide information about the local tissue velocity of deformation, deformation rate results were presented as the root mean square (RMS). Speckle tracking has been validated for force measurements^13,41, 43^. The test–retest reliability of the speckle-tracking analysis method showed strong reliability for all five neck muscles together (ICC 0.79; 95% CI 0.71–0.85), and fair to good reliability for the five muscles separately (ICC 0.43–0.74)^13^.

### Statistical and multivariate data analysis

The variables included in the study were deformation area (% deformation) and deformation rate (% deformation/s) from the five dorsal neck muscles during the tenth neck rotation to the right and to the left.

Three mixed-design analyses of variance (ANOVA) with Bonferroni correction were used to evaluate: (1) Between-subject factor of group and within-group factor of deformation and deformation rate at baseline (two groups, WAD and controls × five muscles: trapezius, splenius, semispinalis capitis, semispinalis cervicis, multifidus), adjusted by sex; (2) Between-subject factor of group and within-group factor of deformation in right rotation between the WAD group after the exercise intervention and the control group baseline data (two groups, WAD three months of data and control baseline data × five muscles: trapezius, splenius, semispinalis capitis, semispinalis cervicis, multifidus); (3) The within-group effect in deformation in rotation to the right after the exercise intervention in the WAD group (two timepoints, baseline, and three-month follow-up × five muscles: trapezius, splenius, semispinalis capitis, semispinalis cervicis, multifidus). In the present study, the two exercise groups (NSE and NSEIT) were analyzed as one exercise group, as the main outcome data from a randomized controlled trial reveal no differences between the NSE and NSEIT groups^24^. For post-hoc contrast analyses, the five neck muscles were divided into three levels: the superficial muscles (trapezius, splenius,), the middle muscle (semispinalis capitis), and the deep muscles (semispinalis cervicis, multifidus). Mauchly’s test of sphericity was used to test the assumption of sphericity in repeated measures. If this assumption was violated (*p* < 0.05), the Greenhouse–Geisser correction was used. *P*-values ≤ 0.05 were considered significant. Effect sizes, partial eta-squared (ηp^2^), were reported, with 0.01 indicating a small effect, 0.06 indicating an intermediate effect, and 0.14 indicating a large effect^54^.

For the left and right neck rotations and the five dorsal muscles, 25 variables comprising deformation areas (total, under curve, over curve, and difference) and deformation rate were assembled as data matrices *X1* and *X2*, sized 194 × 25. Metadata variables (WAD grade, WAD status, age, and sex) were also added to the matrices, to serve as responses or for interpretation. The two matrices were subjected to multivariate techniques principal component analysis, PCA^55^, and orthogonal projections to latent structures discriminant analysis, OPLS-DA^56^, carried out in SIMCA 17.0 (Sartorius Stedim Data Analytics, vs. 17.0, Umeå, Sweden). PCA was used for exploratory analysis of the data, compressing data matrices into a set of principal components, which explained most of the variance in the data. PCA score plots were used to visualize the multi-dimensional data in two dimensions, making it possible to identify outliers and other groupings. OPLS-DA was then used to produce supervised models between the dorsal muscle deformation data and the WAD status. OPLS-DA loading plots were used to identify significant muscle deformations. Here, jack-knifing^57^ was applied during the cross-validation procedure to provide an uncertainty measure for each deformation, with significant terms having confidence intervals not including zero. Leave-out-p cross-validation, where p equaled one seventh of the subjects, was used during the OPLS-DA model calculation.

### Supplementary Information

Supplementary Information 1.

Supplementary Information 2.

## Data Availability

The data sets generated during this study are available from the corresponding author on reasonable request.
