# Supplementary material for: Neck muscle function improves after neck exercises in individuals with whiplash-associated disorders: a case–control ultrasound study with speckle-tracking analyses
Source: Sci Rep. 2024 Aug 13;14:18793. doi: 10.1038/s41598-024-69136-6 (PMC11322392; doi:10.1038/s41598-024-69136-6)
Supplement: Supplementary file 1 — Supplementary Information 1. [file 41598_2024_69136_MOESM1_ESM.pdf]

Supplementary file 1

Table 2. Outcomes in Neck Disability Index (NDI), neck pain and neck muscles fatigue for the WAD and control group at baseline, and at 3 months follow-up, and the change score for the WAD group.

	**WAD**		**Control group (CG)**	**Baseline WAD vs CG**	**Within-group change score WAD**	
	Baseline	3 months	Baseline	P value	Baseline to 3 months	P value
**NDI**	39.1 ± 13.7	31.2 ± 16.1	1.2 ± 1.6	< 0.001	-9.5 ± 8.5	<0.001
**Neck pain before test**	42.0 ± 21.3	22.9 ± 20.2	0.03 ± 0.17	<0.001	-17.9 ± 23.9	<0.001
**Neck pain after test**	42.2 ±21.8	26.4 ± 21.1	0.01 ± 0.08	<0.001	-18.9 ± 25.2	<0.001
**Fatigue before test**	4.0 (3.0 to 6.25)	3.0 (0.5 to 4.0)	0.0 (0.0 to 0.1)	<0.001	-2.0 (0.0 to 3.1)	<0.001
**Fatigue after test**	5.0 (4.0 to 7.0)	3.0 (1.0 to 5.0)	0.0 (0.0 to 0.0)	<0.001	-2.0 (0.9 to 3.2)	<0.001

WAD, whiplash-associated disorders; NDI, Neck Disability Index, scored from 0% (no disability) to 100% (high disability), mean ± standard deviation; Neck pain before and after rotation test, Visual analogue scale, scored from 0 mm (no pain) to 100 mm (worst imaginable pain) mean ± standard deviation; Fatigue neck muscles before and after rotation test Borg CR-10 scale, scored from 0 (no fatigue) to 10 (extremely strong fatigue), median (IQR). Negative values in change score for the WAD group indicate improvements in NDI, pain and fatigue (i.e., less neck related disability, neck pain and neck muscle fatigue).

Table 3. Muscle deformations (% change in length) and deformation rate (% deformation/s) during the tenth (10th) neck rotation to the right and to the left

		WAD baseline		Control baseline		WAD 3 months	
**Neckrotation**		**Right**	**Left**	**Right**	**Left**	**Right**	**Left**
**Test time, seconds**[a]		3.94 ± 0.75	4.09 ± 0.39	4.21 ± 0.27	4.20 ± 0.17	4.15 ± 0.36	4.22 ± 0.20
**Deformation Total area**[b]	TR	5.75 ± 3.14	4.47 ± 3.71	6.83 ± 5.26	4.23 ± 3.04	5.45 ± 3.22	4.46 ± 3.71
	SP	10.08 ± 6.71	8.68 ± 5.75	9.21 ± 8.05	10.34 ± 10.32	10.02 ± 6.88	8.68 ± 5.75
	Scap	14.47 ± 11.71	19.23 ± 14.20	20.83 ± 12.05	14.93 ± 8.46	21.04 ±12.51	19.23 ± 14,20
	Scerv	14.90 ± 10.92	19.14 ± 20.98	25.12 ± 20.91	19.83 ± 15.29	19.08 ± 11.98	19.13 ± 20.97
	MF	13.07 ± 8.65	14.42 ± 11.34	20.01 ± 12.22	14.79 ± 9.41	14.94 ± 10.03	14.42 ± 11.34
**Elongation**	TR	1.52 ± 2.31	3.50 ± 4.18	1.25 ± 2.19	3.29 ± 3.48	1.29 ± 1,51	3.50 ± 4.18
	SP	2.84 ± 3.76	4.42 ± 5.88	4.48 ± 8.47	7.51 ± 10.67	4.76 ± 6.75	4.43 ± 5.88
	Scap	2.74 ± 5.11	12.10 ± 14.99	6.81 ± 12.11	10.92 ± 9.32	3.78 ± 7.24	12.09 ± 14.99
	Scerv	5.21 ± 6.02	13.48 ± 22.29	5.71 ± 9.59	12.63 ± 14.94	4.00 ± 7.50	13.48 ± 22.29
	MF	5.91 ± 7.57	7.94 ± 9.36	3.61 ± 7.51	5.37 ± 7.61	3.17 ± 4.39	7.94 ± 9.36
**Shortening**	TR	4.23 ± 3.74	0.97 ± 1.39	5.58 ± 5.56	0.93 ± 1.23	4.16 ± 3.60	0.97 ± 1.39
	SP	7.23 ± 7.94	4.25 ± 5.67	4.72 ± 5.57	2.82 ± 4.65	5.26 ± 6.81	4.25 ± 5.67
	Scap	11.73 ± 12.32	7.14 ± 9.60	14.02 ± 10.83	4.01 ± 6.09	17.26 ± 14.79	7.1 ± 9.60
	Scerv	9.68 ± 12.09	5.66 ± 8.34	19.41 ± 22.87	7.20 ± 12.98	14.09 ± 14.21	5.65 ± 8.34
	MF	7.15 ± 9.86	6.48 ± 11.60	16.41 ± 14.02	9.43 ± 10.93	14.93 ± 10.03	6.47 ± 11.59
**Deformation rate**							
	TR	1.11 ± 0.49	1.04 ± 0.54	1.17 ± 0.56	0.97 ± 0.51	1.14 ± 0.66	1.05 ± 0.68
	SP	2.42 ± 1.55	1.97 ± 0.89	2.18 ± 0.91	1.90 ± 0.92	2.20 ± 1.16	1.92 ± 0.91
	Scap	2.62 ± 1.08	3.18 ± 1.14	3.12 ± 1.52	2.83 ± 0.89	3.05 ± 1.41	2.88 ± 1.18
	Scerv	3.99 ± 1.79	3.43 ± 1.53	3.73 ± 1.23	3.24 ± 1.30	3.61 ± 1.15	2.94 ± 0.85
	MF	3.19 ± 1.29	3.28 ± 1.67	3.72 ± 1.27	3.52 ± 1.66	3.38 ± 1.47	3.14 ± 1.02

Values are expressed as the mean and standard deviation (SD) for each group, WAD; Whiplash-associated Disorders and Control; Healthy controls.

[a]Test time in seconds for the right and left neck rotation

[b]Total area represents the sum of elongations and shortenings of the muscle.

The five dorsal neck muscles: TR; Trapezius, SP; Splenius, Scap; Semispinalis capitis, Scerv; Semispinalis cervicis and MF; Multifidus
