# Supplementary material for: Neck muscle function improves after neck exercises in individuals with whiplash-associated disorders: a case–control ultrasound study with speckle-tracking analyses
Source: Sci Rep. 2024 Aug 13;14:18793. doi: 10.1038/s41598-024-69136-6 (PMC11322392; doi:10.1038/s41598-024-69136-6)
Supplement: Supplementary file 2 — Supplementary Information 2. [file 41598_2024_69136_MOESM2_ESM.pdf]

Supplementary file 2 for figures 6 and 7

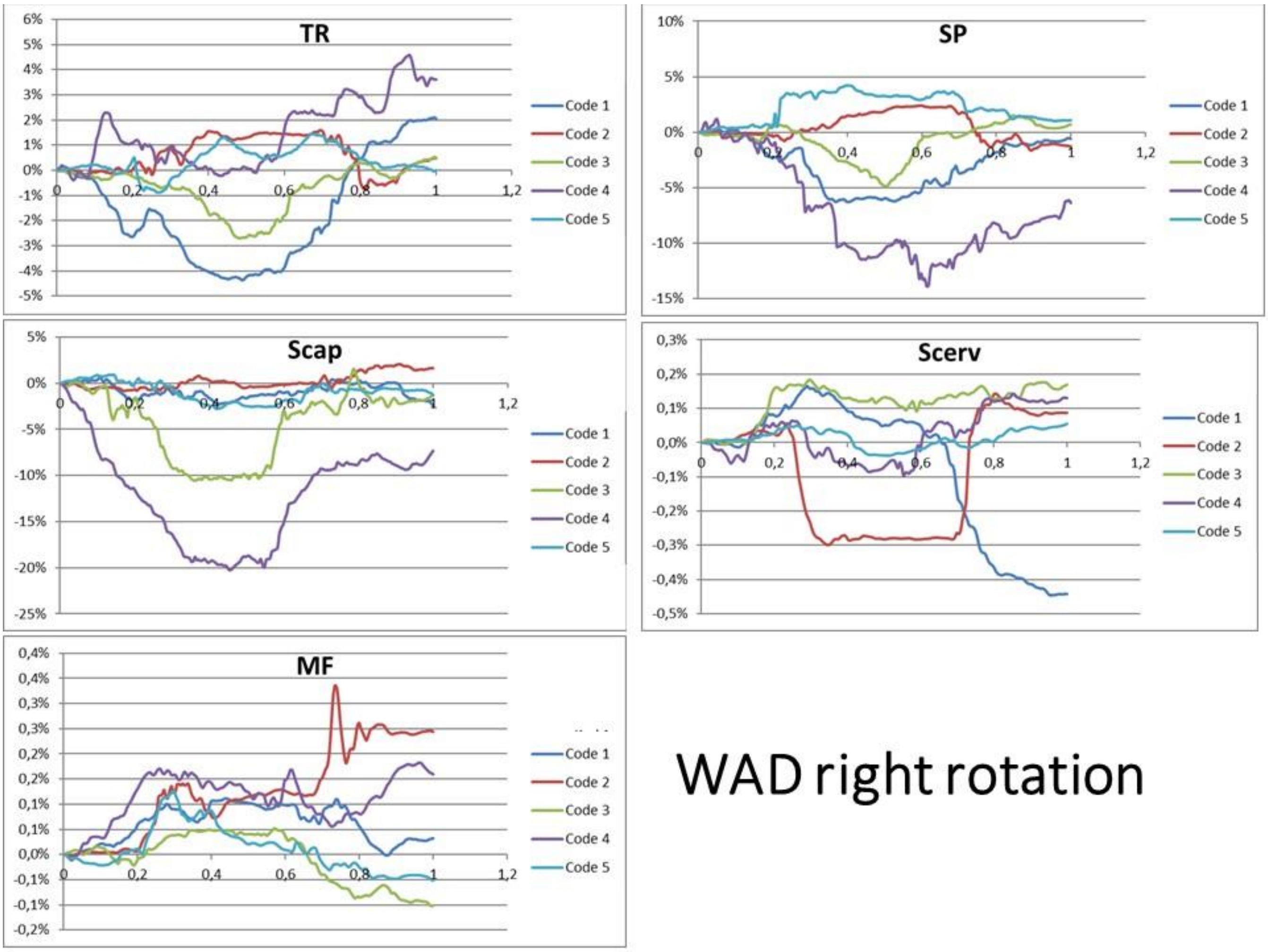

Figure 6a

Figure 6 a-d. Deformation curves in the five dorsal neck muscles in participants with whiplash-associated disorders (WAD) and healthy controls. Codes 1 to 5 represent five individuals with chronic WAD and code 6 to 10 five healthy controls during the tenth neck rotation to the right and left.

a) right rotation in WAD, b) right rotation in controls, c) left rotation in WAD, d) left rotation in controls; TR: Trapezius; SP: Splenius; Scap: Semispinalis capitis; Scerv: Semispinalis cervicis; and MF: Multifidus

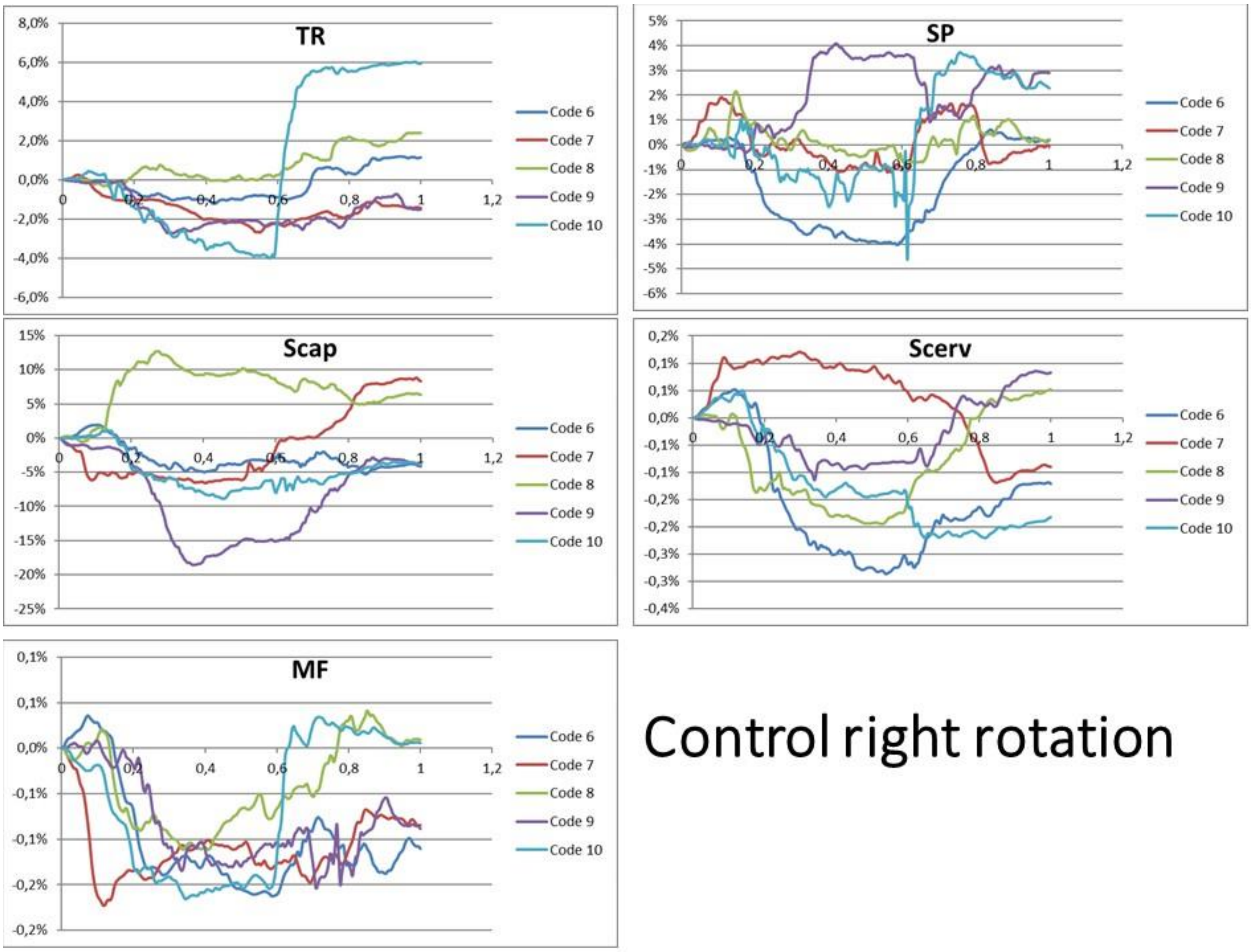
TR
Code 6
Code 7
Code 8
Code 9
Code 10
SP
Code 6
Code 7
Code 8
Code 9
Code 10
Scap
Code 6
Code 7
Code 8
Code 9
Code 10
Scerv
Code 6
Code 7
Code 8
Code 9
Code 10
MF
Code 6
Code 7
Code 8
Code 9
Code 10
Control right rotation

Figure 6b

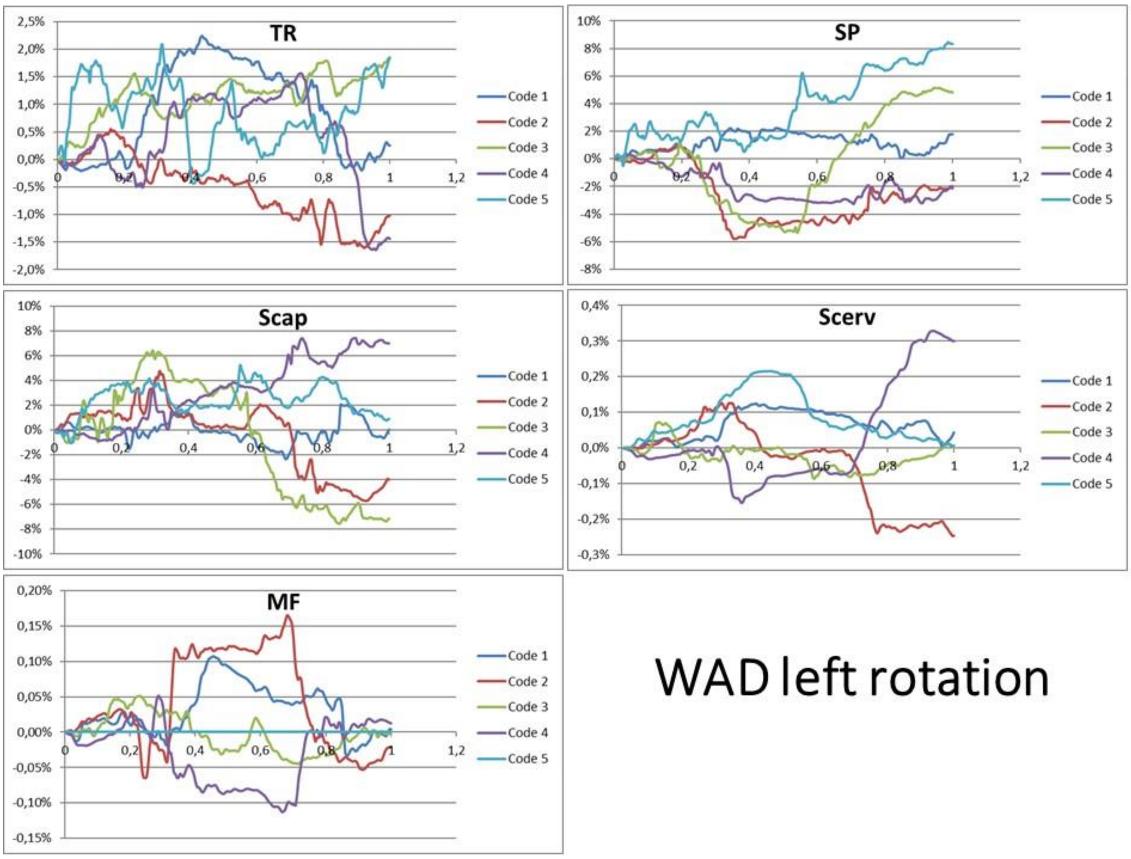

TR
2,5%
2,0%
1,5%
1,0%
0,5%
0,0%
-0,5%
-1,0%
-1,5%
-2,0%
0
0,2
0,4
0,6
0,8
1
1,2
Code 1
Code 2
Code 3
Code 4
Code 5
SP
10%
8%
6%
4%
2%
0%
-2%
-4%
-6%
-8%
0
0,2
0,4
0,6
0,8
1
1,2
Code 1
Code 2
Code 3
Code 4
Code 5
Scap
10%
8%
6%
4%
2%
0%
-2%
-4%
-6%
-8%
-10%
0
0,2
0,4
0,6
0,8
1
1,2
Code 1
Code 2
Code 3
Code 4
Code 5
Scerv
0,4%
0,3%
0,2%
0,1%
0,0%
-0,1%
-0,2%
-0,3%
0
0,2
0,4
0,6
0,8
1
1,2
Code 1
Code 2
Code 3
Code 4
Code 5
MF
0,20%
0,15%
0,10%
0,05%
0,00%
-0,05%
-0,10%
-0,15%
0
0,2
0,4
0,6
0,8
1
1,2
Code 1
Code 2
Code 3
Code 4
Code 5
WAD left rotation

Figure 6c

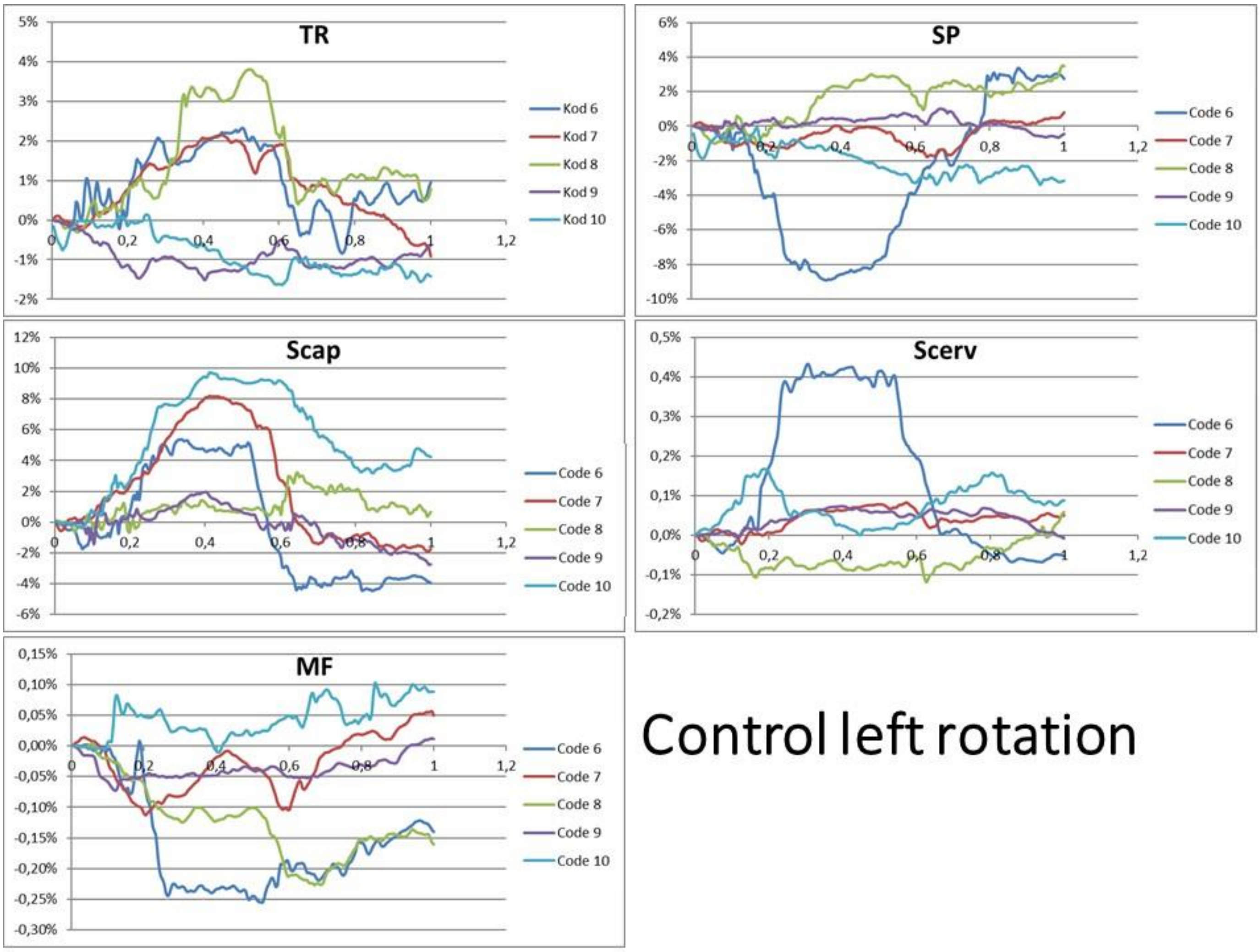
TR
5%
4%
3%
2%
1%
0%
-1%
-2%
0
0,2
0,4
0,6
0,8
1
1,2
Kod 6
Kod 7
Kod 8
Kod 9
Kod 10
SP
6%
4%
2%
0%
-2%
-4%
-6%
-8%
-10%
Code 6
Code 7
Code 8
Code 9
Code 10
Scap
12%
10%
8%
6%
4%
2%
0%
-2%
-4%
-6%
Code 6
Code 7
Code 8
Code 9
Code 10
Scerv
0,5%
0,4%
0,3%
0,2%
0,1%
0,0%
-0,1%
-0,2%
Code 6
Code 7
Code 8
Code 9
Code 10
MF
0,15%
0,10%
0,05%
0,00%
-0,05%
-0,10%
-0,15%
-0,20%
-0,25%
-0,30%
Code 6
Code 7
Code 8
Code 9
Code 10
Control left rotation

Figure 6d

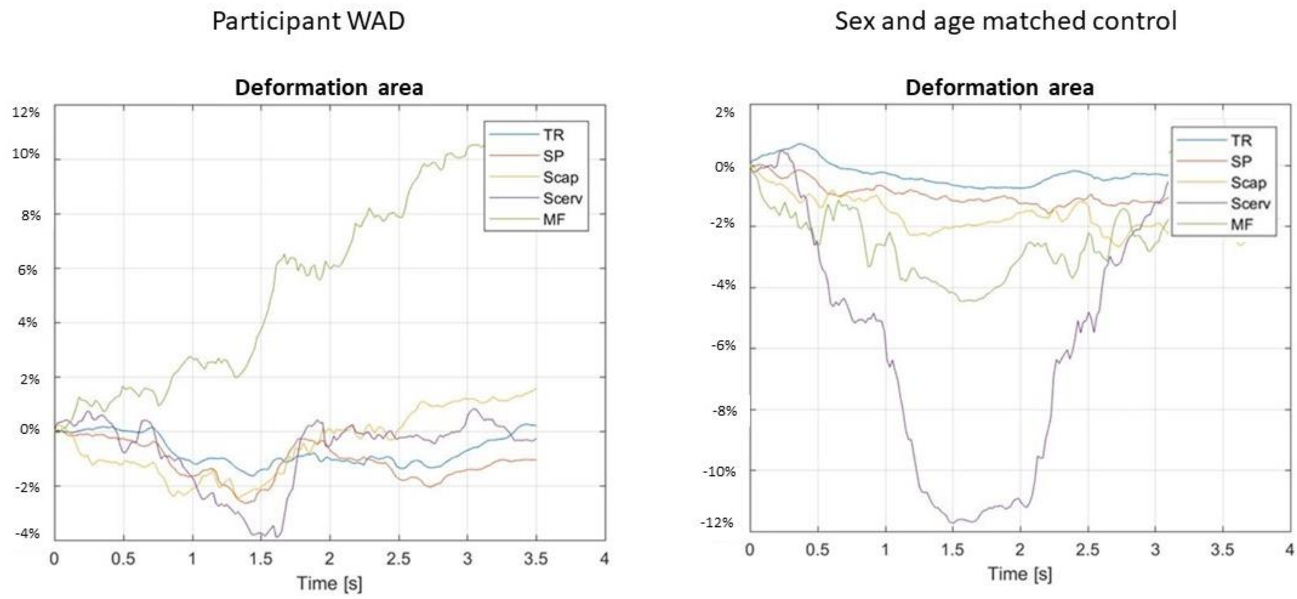

Figure 7. This diagram illustrates muscle deformation sequences during neck rotation to the right for the two different patterns of muscle deformation areas for one participant with WAD, and for one pain free participant, sex and age matched healthy control. A line represents the changes observed in the ROI (deformation %) in one muscle, during 20 degrees right rotation. Muscle shortening is the region (area) below zero (negative values) and muscle elongation represents the region (area) above zero (positive values). The sum of negative and positive areas represents the total muscle deformation during one neck rotation, from neutral position of the head 20 degrees and back to midline. When the line crosses 0%, the muscle deformation shifts from shortening to elongation, or vice versa. (TR: Trapezius; SP: Splenius; Scap: Semispinalis capitis; Scerv: Semispinalis cervicis; and MF: Multifidus)
